# Fermented jellyfish (*Rhopilema esculentum*) collagen enhances antioxidant activity and cartilage protection on surgically induced osteoarthritis in obese rats

**DOI:** 10.3389/fphar.2023.1117893

**Published:** 2023-01-30

**Authors:** Sabri Sudirman, Chun-Yu Chen, Chun-Kai Chen, Jerrell Felim, Hsiang-Ping Kuo, Zwe-Ling Kong

**Affiliations:** ^1^ Fisheries Product Technology, Faculty of Agriculture, Universitas Sriwijaya, Indralaya, Indonesia; ^2^ Department of Food Science, National Taiwan Ocean University, Keelung, Taiwan

**Keywords:** *Bacillus subtilis* natto, collagen, jellyfish, obesity, osteoarthritis

## Abstract

Collagen has been considered a key treatment option in preventing damage to the articular cartilage over time and supporting the healing process, following the onset of osteoarthritis (OA). This study aimed to investigate the effect of collagen fermented from jellyfish (FJC) by *Bacillus subtilis* natto on anterior cruciate ligament transection with medial meniscectomy (ACLT + MMx)-induced knee OA in high-fat diet (HFD)-induced obesity in rats. The male Sprague–Dawley rats were fed an HFD for 6 weeks before ACLT + MMx surgery, after which they were administered a daily oral gavage of saline (control, OA, and OBOA), either with FJC (20 mg/kg, 40 mg/kg, and 100 mg/kg body weight) or glucosamine sulfate as a positive control (GS; 200 mg/kg body weight) for 6 weeks. Treatment with FJC decreased the fat weight, triglyceride, and total cholesterol levels in obese rats. Additionally, FJC downregulated the expression of some proinflammatory cytokines, including tumor necrosis factor-α, cyclooxygenase-2, and nitric oxide; suppressed leptin and adiponectin expression; and attenuated cartilage degradation. It also decreased the activities of matrix metalloproteinase (MMP)-1 and MMP-3. These results demonstrated that FJC showed a protective effect on articular cartilage and also suppressed the degradation of cartilage in an animal OA model, suggesting its potential efficacy as a promising candidate for OA treatment.

## 1 Introduction

Osteoarthritis (OA) is a degenerative disease of joint instability, and more than 240 million people worldwide suffer from this disease (Lee et al., 2020; Katz et al., 2021). The main features of OA include progressive erosion of articular cartilage, subchondral bone sclerosis, and synovitis (Feng et al., 2017). In the treatment of OA, it is always a challenge to seek effective therapies to reduce joint degeneration, improve joint mobility, and relieve joint pain. The current treatments for mild to moderate OA include acetaminophen and non-steroidal anti-inflammatory drugs (NSAIDs), but these drugs have potential adverse effects on the gastrointestinal tract, liver, heart, and kidney and will aggravate the reaction as the dose and treatment time increase (McAlindon et al., 2014). Therefore, it is necessary to find an alternative therapy for OA such as by using natural products either as functional foods or food supplements.

Jellyfish (*Rhopilema esculentum*) has been an indispensable ingredient in Chinese cuisine for more than a thousand years. Collagen has been identified as one of the chemical compositions of jellyfish (Khong et al., 2016). Collagen is a major structural protein in bone and animal skin (Gistelinck et al., 2016). Collagen is composed of glycine, proline, and proline amino acids (Albaugh et al., 2017). Collagen is a major component in the extracellular matrix and also makes up the cartilage in humans, especially collagen type II (Amirrah et al., 2022). A previous study reported that collagen is widely used in the pharmaceutical, biomedical, and film industries (Benjakul et al., 2010). A previous study also reported that collagen effectively improves OA symptoms by decreasing both the total VAS score and WOMAC Index (García-Coronado et al., 2018).

In the case of OA treatment, collagen supplementation is also considered to be a key treatment option in preventing damage to articular cartilage over time and supporting the healing process after OA. However, due to insufficient bone and leather supply, collagen prices have gradually increased, and religious factors have further restricted the use of pigs and cattle (Kaewdang et al., 2014), whereas porcine pepsin has been commonly used for collagen production (Benjakul et al., 2010). According to these conditions, the research for alternative collagen sources has been a major issue. The alternative sources include marine animals and the use of bacterial enzymes as an alternative to replacing porcine pepsin during collagen extraction. A previous study reported that *Bacillus subtilis* natto has been used in fermented collagen production (Hsu and Chiang, 2009). Therefore, this study aimed to investigate the ameliorative effects of fermented jellyfish (*Rhopilema esculentum*) collagen (FJC) on anterior cruciate ligament transection with meniscectomy-induced OA in high-fat diet-induced obese rats.

## 2 Materials and methods

### 2.1 Materials

Jellyfish (*Rhopilema esculentum*) was obtained from Fuzhou Zelin Food Co. Ltd., (Fuzhou, China). Glucosamine sulfate was purchased from Chen Ta Plama Mfg, Co. Ltd., (Tainan, Taiwan). The standard laboratory chow-fed diet (Laboratory Rodent Diet 5,001) was purchased from PMI Nutrition International, Inc. (Brentwood, MO, United States). Lard was purchased from MP Biomedicals (Cat. No. 902140, Santa Ana, CA, United States). A total cholesterol commercial kit (Ref. CH7945) was purchased from Randox Laboratories, Ltd., (Crumlin, United Kingdom), and triglyceride (TG; Cat. No. ETGA-200) and high-density lipoprotein cholesterol (HDL-C; Cat. No. EHDL-100) kits were purchased from BioAssay Systems (Hayward, CA, United States of America). MMP-1 (Cat. No. E-EL-R0617), MMP-3 (Cat. No. E-EL-R0619), and prostaglandin (PG)-E2 (Cat. No. E-EL-0034) ELISA kits were purchased from Elabscience Biotechnology Inc. (Houston, TX, United States). Interleukin (IL)-1β (Cat. No. SEA563Ra), TNF-α (Cat. No. SEA133Ra), and leptin (Cat. No. SEA084Ra) ELISA kits were purchased from USCN Life Science Inc. (Wuhan, China). NF-κB (Cat. No. ER1186) ELISA kits were purchased from Wuhan Fine Biotech Co., Ltd., (Wuhan, China). Zoletil 50 and Lofalin injections (cefazolin sodium) were purchased from Virbac (Carros, France) and Gentle Pharm Co. Ltd., (Yunlin, Taiwan), respectively. Formaldehyde solution was purchased from Avantor Performance Materials Inc. (Radnor, PA, United States).

### 2.2 Jellyfish collagen (JC) and fermented jellyfish collagen (FJC) extraction

The jellyfish collagen extract was obtained according to the previous method (Khong et al., 2018). Briefly, jellyfish was washed with distilled water and extracted with 0.1 M NaOH. After homogenization, it was extracted with 0.5 M acetic, and ultrasound was performed for 2 h and then rigorously mixed at 4°C for 2 h. The supernatant was collected by centrifugation (10,000 × g, 4°C, 2 h), and then, NaCl was added to a final concentration of 4.5 M. The precipitate was collected by centrifugation (10,000 × g, 4°C, 2 h) and dissolved in 0.5 M acetic acid. The solution was dialyzed against distilled water in a dialysis bag for 3 days and then lyophilized to obtain the jellyfish collagen extract. FJC was obtained according to the previous method (Hsu and Chiang, 2009). Briefly, JC was added to dH_*2*_O, glucose (2%), and bacterial fluids (containing 2% of *Bacillus subtilis* natto culture) and cultivated at 37°C and shaken at 120 rpm for 3 days. After incubation time, the mixture was centrifuged at 10,000 × g for 25 min, filtered by using a filter paper, and freeze-dried to obtain FJC and was then stored at −20°C for further analysis.

### 2.3 Dried jellyfish and JFC characterization

The proximate composition of dried jellyfish was measured according to the Association of Official Analytical Chemists (AOAC) method (AOAC, 2000). Briefly, the moisture content of jellyfish was determined by oven drying at 105°C to obtain constant dry weight. The ash was measured by complete incineration of the JC at 550°C in a muffle furnace (AOAC method 930.05). The fat from jellyfish was extracted using a Soxhlet extractor for 6 h with ethyl ether (AOAC method 991.36). Total nitrogen was analyzed by the Kjeldahl method, whereas the protein content was measured by multiplying total nitrogen by a factor of 6.25 (AOAC method 981.10). The carbohydrate content was determined by the difference method.

The amino acid composition of FJC was measured by the high-performance liquid chromatography (HPLC) method, whereas the functional group of FJC was determined by Fourier transform-infrared (FT-IR) spectroscopy (Bruker-Tensor II, Massachusetts, United States), according to the previous method (Camacho et al., 2001). Additionally, the UV-Vis absorbance of fermented jellyfish collagen was also detected, according to the previous method (Seixas et al., 2020).

### 2.4 Animal model

**FIGURE 1:**
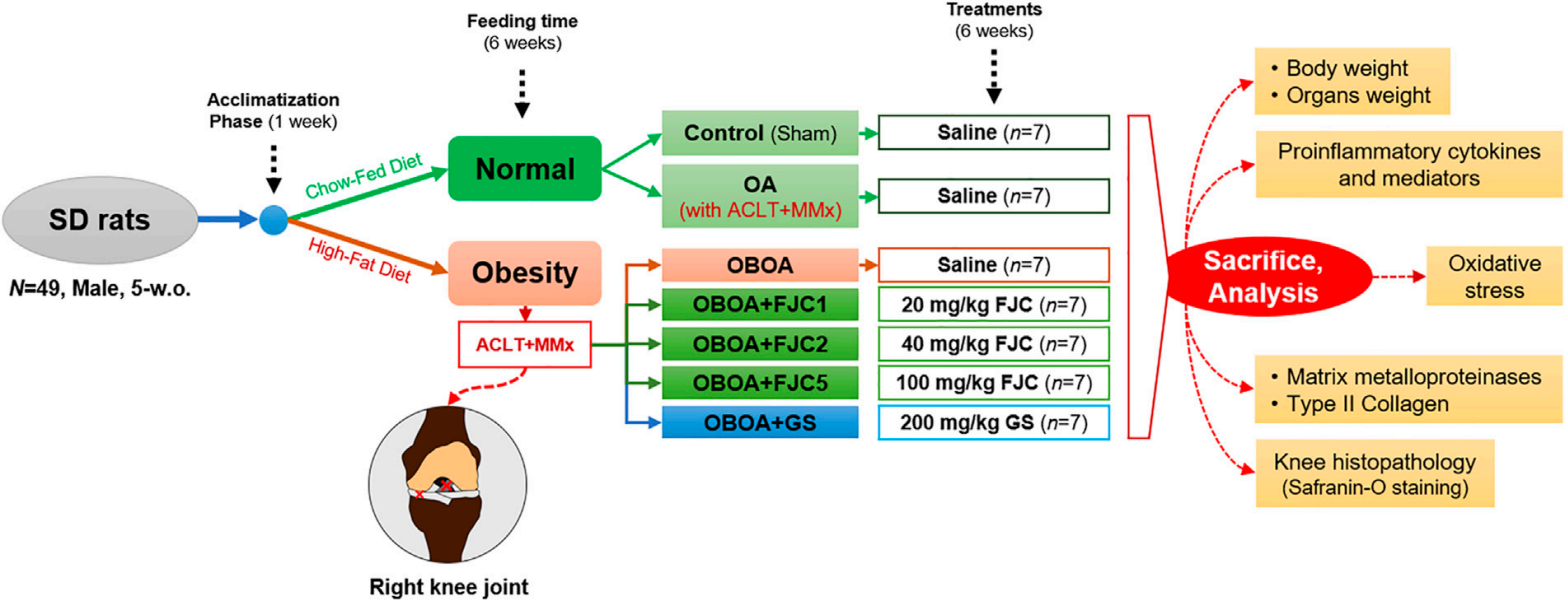
Flowchart of fermented jellyfish collagen (FJC) treatment on anterior cruciate ligament transection with medial meniscectomy (ACLT + MMx)-induced osteoarthritis (OA) in high-fat diet-induced obese (OB) rats.

Male Sprague–Dawley (SD) rats (5 weeks old) were purchased from BioLASCO Co. Ltd., (Yilan, Taiwan), then housed individually, and maintained at 25°C ± 2°C with 50% ± 10% of humidity under a 12-h light/dark cycle throughout the experiments. The Institutional Animal Care and Use Committee (IACUC Approval No. 109003) of National Taiwan Ocean University reviewed and approved all protocols. The rats (*N* = 49) were acclimatized for 1 week and administered a standard chow-fed diet (Laboratory Rodent Diet 5,001) and water *ad libitum*. After acclimatization, the rats were randomly divided into two main groups: the normal group [*n* = 14; fed with chow-fed diet (CFD)] and the obesity (OB) group [*n* = 35; fed with high-fat diet (HFD)] (Figure 1). The HFD is composed of ∼20% of fat in the total diet or ∼40% of calories from fat. After 6 weeks of feeding time, the normal group was subdivided into two subgroups: the sham group (control, without ACLT + MMx surgery, *n* = 7) and the OA group (with ACLT + MMx surgery, *n* = 7), whereas the OB group was subdivided into five subgroups (*n* = 7) and received ACLT + MMx surgery (OBOA groups). Four of the OBOA groups were administered a daily oral gavage with one of three different doses of FJC [OBOA + FJC1: 20 mg/kg body weight; OBOA + FJC2: 40 mg/kg body weight; FJC5: OBOA + 100 mg/kg body weight] or a dose of glucosamine sulfate as a positive control (OBOA + GS: 100 mg/kg body weight), according to the previous study with a modification (Löscher, 2007; Zhuang et al., 2012). These doses are the effective amount of collagen for human intake, according to the human equivalent dose (HED) formula of about 2.5 g–15 g/day (Nair and Jacob, 2016; Paul et al., 2019). The final OBOA group was orally administered saline (OBOA). The rats were euthanized by exposure to carbon dioxide (CO_2_) in an empty chamber after 6 weeks of treatments. The rats fasted for 12 h before surgery and sacrifice, and on the day of sacrifice, the body weight and adipose tissues were measured using a weighing scale. Whole blood and the operated knee joint were collected and stored for further analysis.

### 2.5 Surgically induced OA

The surgically induced OA by ACLT + MMx surgery was performed, according to the previous method (Hayami et al., 2006). Briefly, the rats were anesthetized by intraperitoneal injection of Zoletil (25 mg/kg body weight), and hair on the right knee was shaved using a digital hair clipper. In the control groups, surgery was performed by opening the knee-joint capsule without ACLT + MMx (sham), whereas the OBOA groups (OBOA, OBOA + FJC1, OBOA + FJC2, OBOA + FJC5, and GS) underwent ACLT + MMx. The knee-joint capsule and skin were closed by sewing with chromic catgut sterile (4-0) and silk braided sterile sutures (3-0; Unik Surgical Sutures Mfg, Co. Ltd., Taipei, Taiwan), respectively. The rats were then intraperitoneally injected with cefazolin antibiotic (30 mg/kg) for 3 days after surgery to prevent surgery-related infection.

### 2.6 Blood sample collection

The whole blood of rats was collected from the abdominal aorta of the rats on the day of sacrifice using a heparinized syringe. The plasma or serum was separated from whole blood by centrifugation (Kubota Centrifuge 3,500; Kubota Corp., Tokyo, Japan) at 3,000 rpm and 4°C for 15 min. The supernatant was gently collected using a micropipette and stored at −20°C for future analysis (Sudirman et al., 2019).

### 2.7 Lipid properties and inflammatory cytokine analysis

The triglycerides, total cholesterol, and high-density lipoprotein cholesterol in blood serum were analyzed using commercial kits. The inflammatory markers, including tumor necrosis factor (TNF)-α, interleukin (IL)-1β, leptin, matrix metalloproteinase (MMP)-1 and MMP-13, and prostaglandin (PG)-E2, were analyzed using enzyme-linked immunosorbent assay (ELISA kits). The analysis was performed according to manufacturer’s instructions.

### 2.9 Knee histopathology staining

The operated knee joints were collected on the day of sacrifice and fixed with a 4% formaldehyde solution. After the decalcification process, the paraffin-embedded sections (5 μm) were stained with Safranin-O. This staining was performed by Li Pie Co. Ltd., (Taichung, Taiwan).

### 2.10 Statistical analysis

All data were expressed as the mean ± standard deviation (SD) and analyzed by one-way ANOVA with Duncan’s *post hoc* test (*p* < 0.05) using SPSS (v.22.0; IBM Corp., Armonk, NY, United States). All graphics were produced using GraphPad Prism 5.0 software (GraphPad Software, Inc., San Diego, CA, United States).

## 3 Results

### 3.1 Chemical composition of dried jellyfish and fermented jellyfish collagen

**FIGURE 2:**
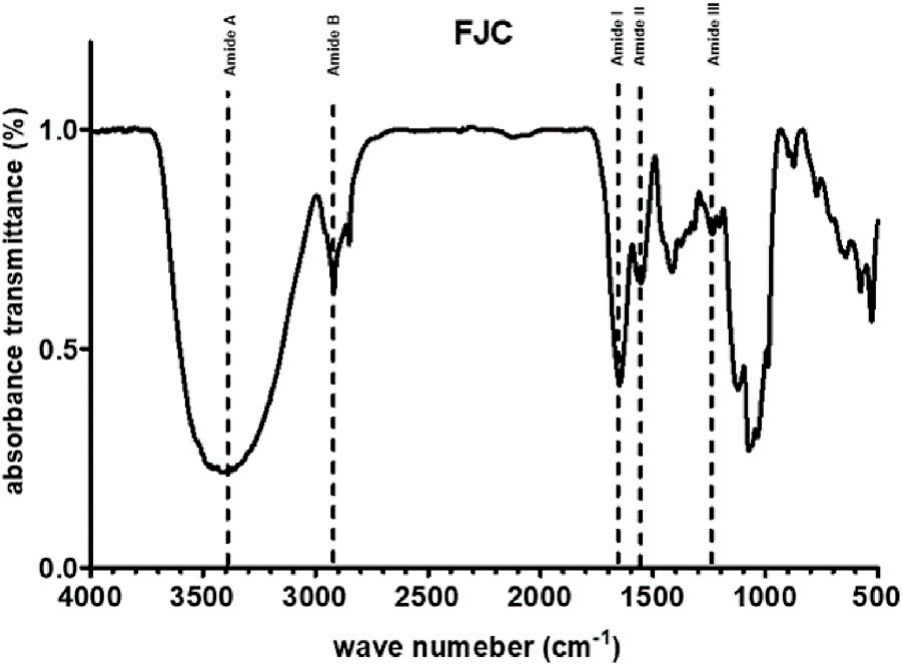
Fourier transform-infrared (FT-IR) spectra of fermented jellyfish collagen (FJC).

**FIGURE 3:**
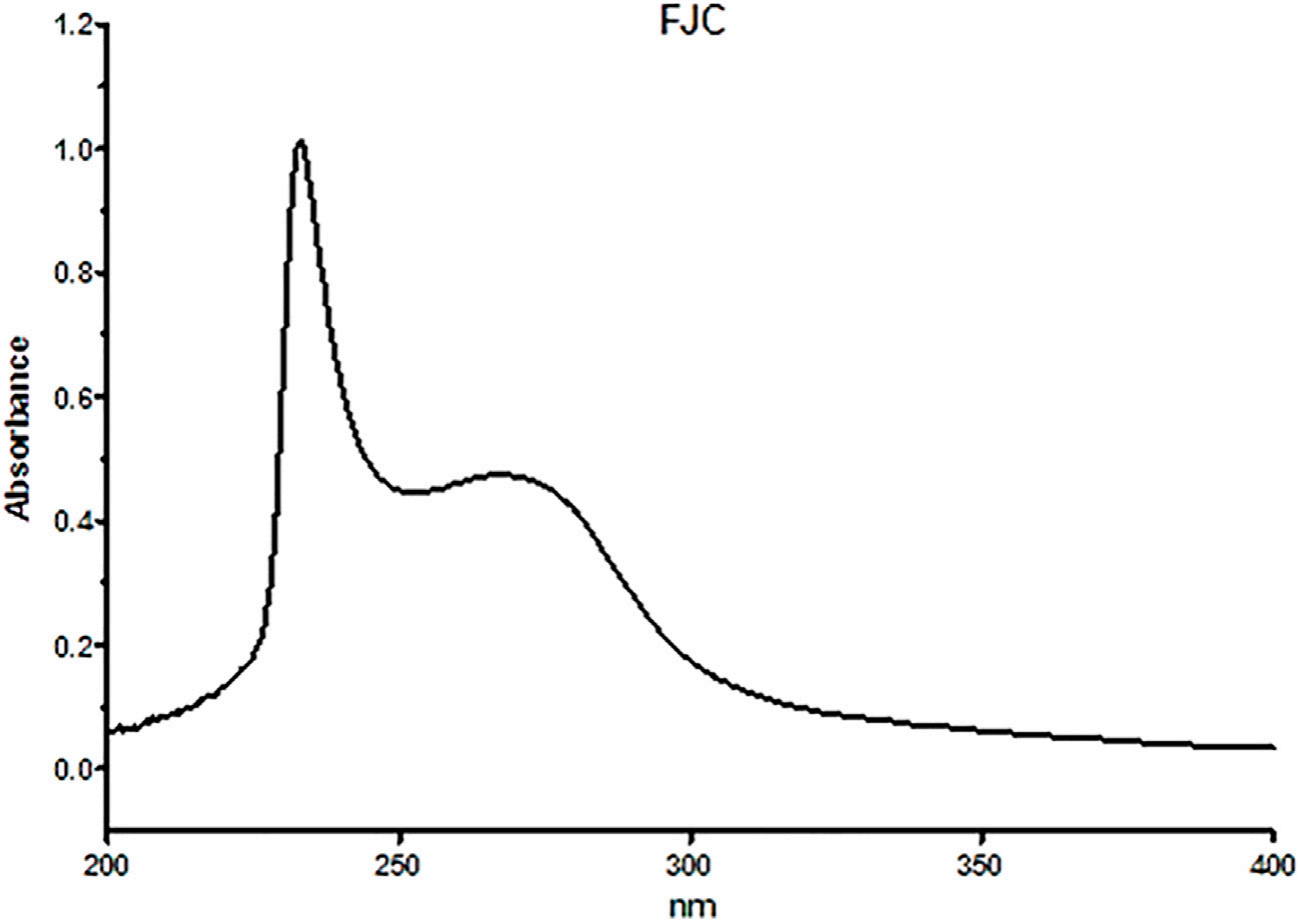
UV-Visible absorption of fermented jellyfish collagen (FJC).

Table 1 shows that freeze-dried jellyfish are composed of high ash (81.53%) and protein (10.27%). Jellyfish is also composed of low levels of carbohydrates, water, and lipid. According to the HPLC method, the fermented jellyfish collagen (FJC) shows a high level of glycine (324.84 residues) and also presents proline (95.63 residues) and hydroxyproline (46.86 residues) amino acids (Table 2). The functional groups by FT-IR and collagen identification by UV-Visible spectroscopy of FJC’s collagen are shown in Figure 2 and Figure 3, respectively. Figure 2 shows the five amide peaks that were detected in the collagen: amide A (3,400 cm^−1^–3,440 cm^−1^), amide B (2,900 cm^−1^), amide I (1,600 cm^−1^–1700 cm^−1^), amide II (∼1,550 cm^−1^), and amide III (1,220 cm^−1^–1,320 cm^−1^). The maximum UV radiation of the FJC is around 233 nm, as shown in Figure 3.

**TABLE 1:** Proximate composition of freeze-dried jellyfish.

Component	Values ± SD (% dry weight)
Moisture	2.92 ± 0.05
Protein	10.27 ± 0.09
Lipid	0.15 ± 0.13
Ash	81.53 ± 0.29
Carbohydrates	5.13

Data are shown as the mean ± SD (*n* = 3).

**TABLE 2:** Amino acid compositions of fermented jellyfish collagen (JFC).

Amino acid	Residues/1,000 residues
Glycine	324.84
Glutamic acid	103.21
Proline	95.63
Alanine	100.74
Aspartic acid	76.86
Arginine	55.87
Hydroxyproline	46.86
Serine	29.85
Lysine	30.22
Threonine	27.33
Leucine	30.68
Valine	22.21
Isoleucine	11.78
Phenylalanine	14.86
Methionine	8.57
Tyrosine	7.23
Cysteine	2.40

### 3.2 FJC supplementation reduces adipose tissue weight

Table 3 shows that a high dose of FJC supplementation (OBOA + FJC5) significantly (*p* < 0.05) reduced the epididymal adipose fat when compared to without treatment (OBOA). It also significantly (*p* < 0.05) decreased abdominal fat. Additionally, supplementation of FJC in high doses also significantly (*p* < 0.05) reduced the liver and kidney weights when compared to the OA group.

**TABLE 3:** Effects of fermented jellyfish collagen (FJC) and glucosamine sulfate (GS) on the change in organ and adipose tissue weights in rats after 6 weeks of treatment.

Group	Organ weights (% of body weight)			
	Liver	Kidney	Epididymal fat	Abdominal fat
Control	3.04 ± 0.22^abc^	0.76 ± 0.03^c^	1.68 ± 0.29^bc^	1.87 ± 0.45^cd^
OA	3.18 ± 0.15^c^	1.39 ± 0.37^b^	1.39 ± 0.37^c^	1.89 ± 0.63^d^
OBOA	2.93 ± 0.22^abc^	2.20 ± 0.60^a^	2.20 ± 0.60^a^	2.91 ± 0.46^a^
OBOA + FJC1	3.09 ± 0.38^bc^	2.04 ± 0.38^a^	2.04 ± 0.38^ab^	2.51 ± 0.39^abc^
OBOA + FJC2	2.85 ± 0.21^ab^	0.72 ± 0.05^c^	1.84 ± 0.38^ab^	2.25 ± 0.55^bcd^
OBOA + FJC5	2.80 ± 0.17^a^	0.75 ± 0.07^c^	1.65 ± 0.26^bc^	1.94 ± 0.22^cd^
OBOA + GS	2.89 ± 0.23^ab^	0.68 ± 0.07^c^	1.89 ± 0.36^ab^	2.71 ± 0.67^ab^

Data represent the mean ± SD (*n* = 7). Different letters (a–d) indicate statistically different values (*p*< 0.05) between groups, as analyzed by using one-way ANOVA with Duncan’s *post hoc* test.

### 3.3 FJC supplementation regulates triglyceride and cholesterol levels

**FIGURE 4:**
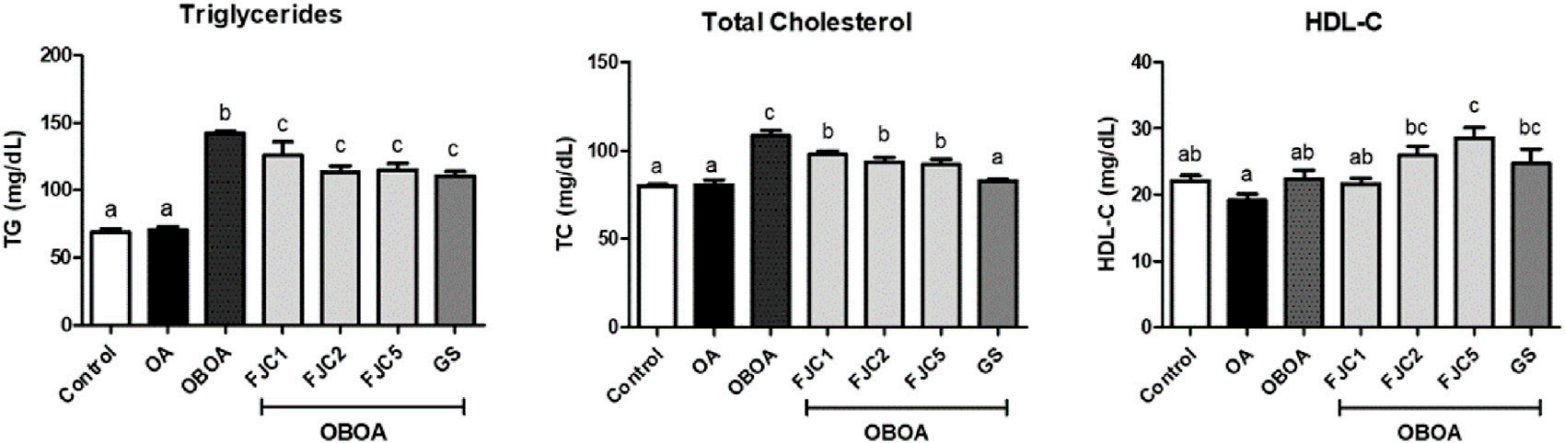
Effects of fermented jellyfish collagen (FJC) and glucosamine sulfate (GS) on triglycerides, total cholesterol level, and high-density lipoprotein cholesterol (HDL-C) in rats’ plasma after treatment for 6 weeks. Data represent the mean ± SD (*n* = 7). Different letters indicate (a–c) statistically different values (*p* < 0.05) between groups determined using one-way ANOVA with Duncan’s *post hoc* test.

High levels of triglycerides (TG) and total cholesterol (TC) were detected in the OBOA group (Figure 4). However, the OBOA group showed low-level high-density lipoprotein cholesterol (HDL-C). Oral supplementation of FJC for 6 weeks significantly (*p* < 0.05) reduced the levels of TG and TC, and a high dose of FJC (OBOA + FJC5) treatment significantly (*p* < 0.05) elevated the HDL-C level.

### 3.4 FJC supplementation enhances antioxidant activities and reduces pro-inflammatory mediators and cytokines

**FIGURE 5:**
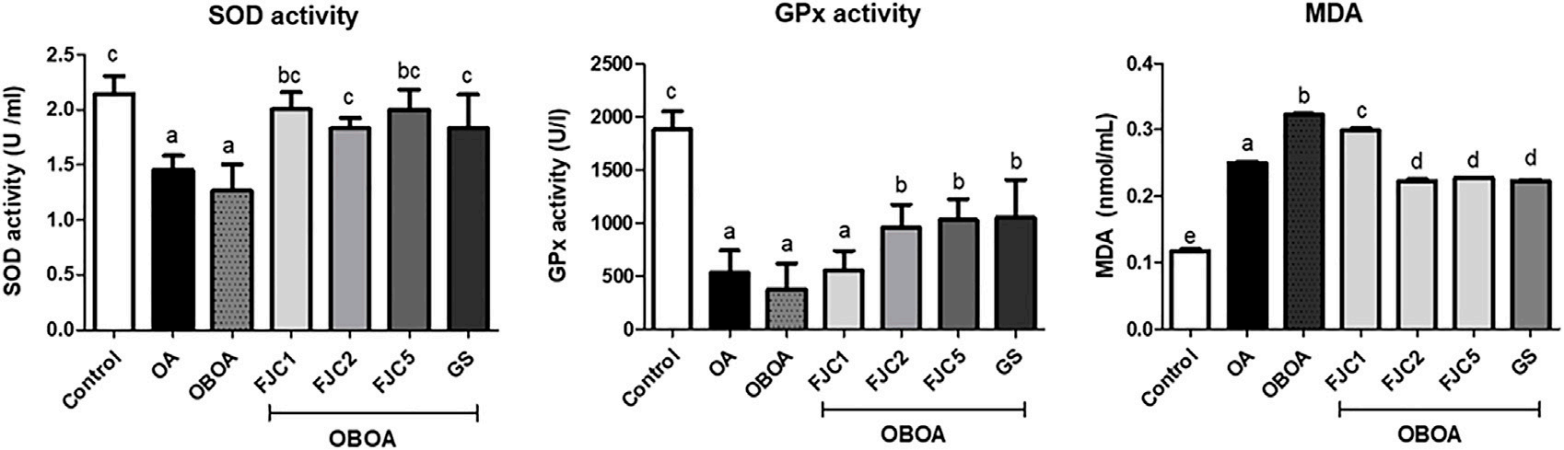
Effects of fermented jellyfish collagen (FJC) and glucosamine sulfate (GS) on superoxide dismutase (SOD) activity, glutathione peroxidase (GPx), and malondialdehyde (MDA) levels in rats’ plasma after treatment for 6 weeks. Data represent the mean ± SD (*n* = 7). Different letters indicate (a–d) statistically different values (*p* < 0.05) between groups determined using one-way ANOVA with Duncan’s *post hoc* test.

**FIGURE 6:**
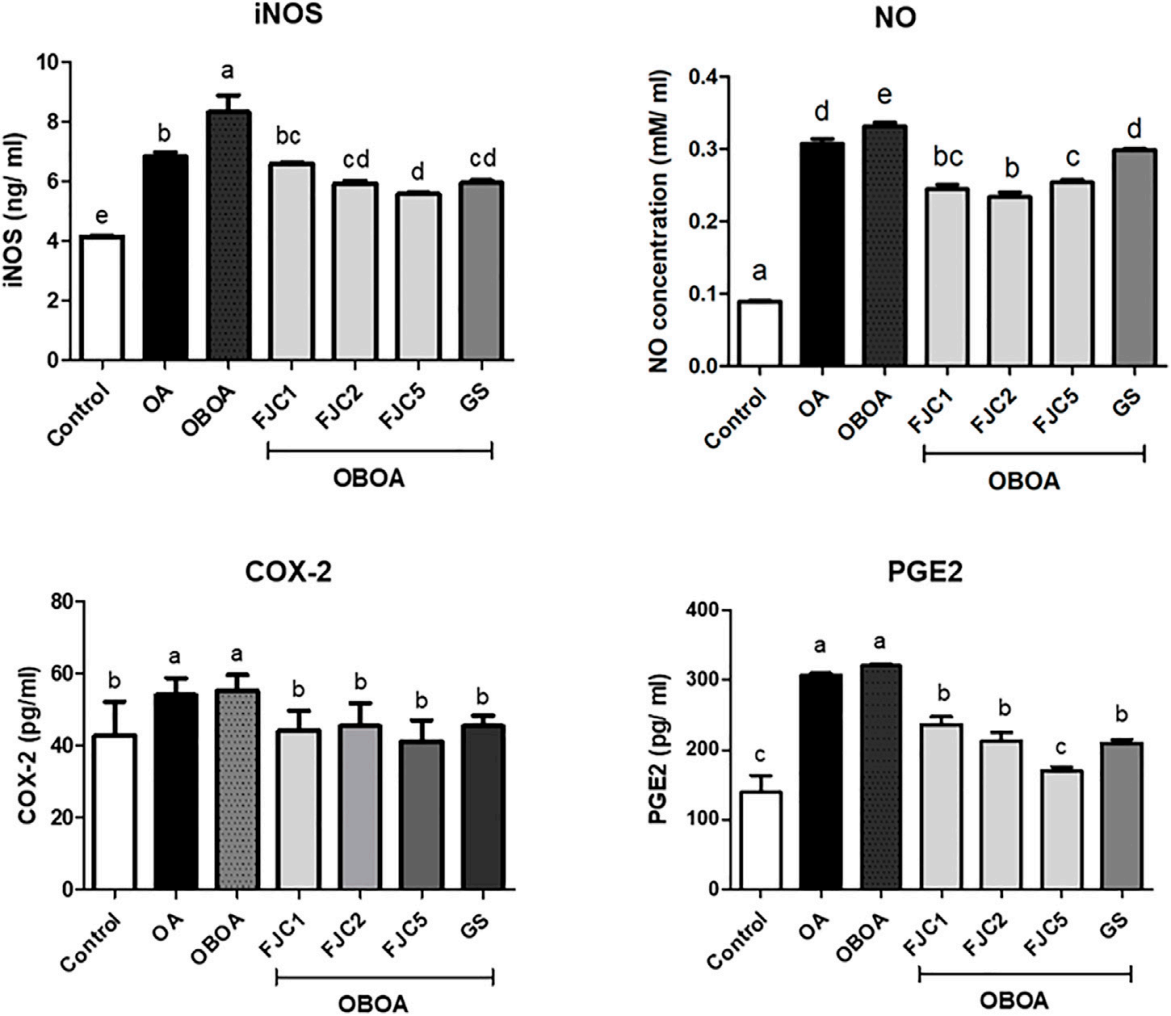
Effects of fermented jellyfish collagen (FJC) and glucosamine sulfate (GS) on inducible nitric oxide synthase (iNOS), nitric oxide (NO), cyclooxygenase (COX)-2, and prostaglandin E2 (PGE2) in rats’ plasma after treatment for 6 weeks. Data are shown as the mean ± SD (*n* = 7). Different letters indicate (a–e) statistically different values (*p* < 0.05) between groups determined using one-way ANOVA with Duncan’s *post hoc* test.

**FIGURE 7:**
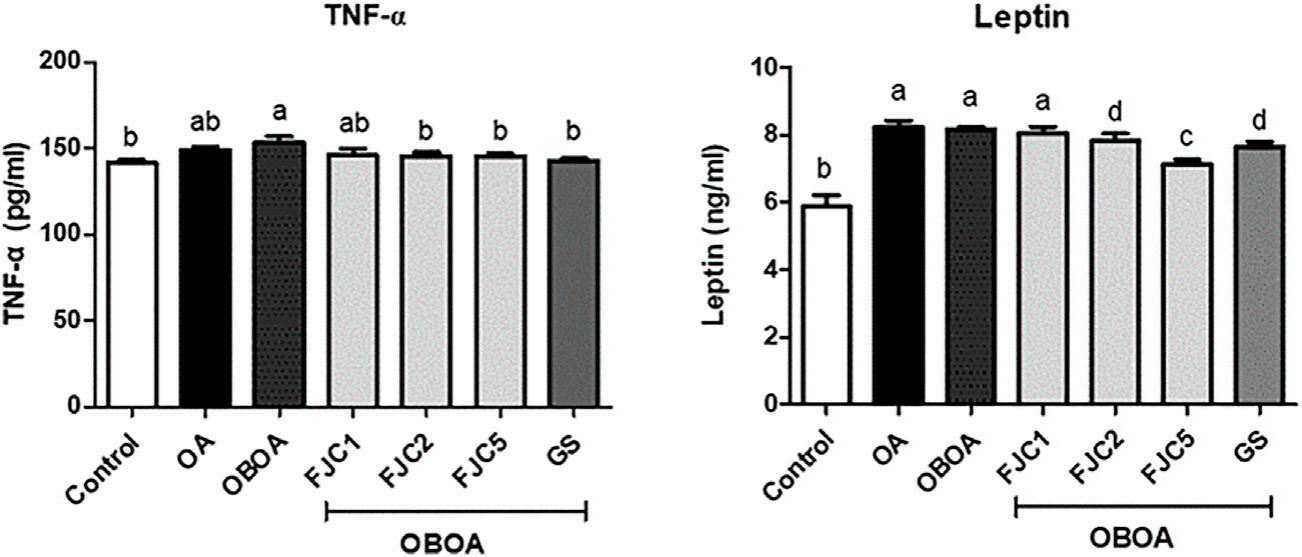
Effects of fermented jellyfish collagen (FJC) and glucosamine sulfate (GS) on tumor necrosis factor-alpha (TNF-α), leptin, and adiponectin in rats’ plasma after treatment for 6 weeks. Data represent the mean ± SD (*n* = 7). Different letters indicate (a–d) statistically different values (*p* < 0.05) between groups determined using one-way ANOVA with Duncan’s *post hoc* test.

The OA and OBOA groups showed low antioxidant activities, such as superoxide dismutase (SOD) and glutathione peroxidase (GPx), as shown in Figure 5. As a result, these groups possessed high malondialdehyde (MDA) levels. After 6 weeks of treatment, medium and high doses of FJC (OBAO + FJC2 and OBAOFJC5, respectively) significantly (*p* < 0.05) enhanced the SOD and GPX activities. Additionally, the MDA level significantly (*p* < 0.05) decreased after being treated with FJC. The concentration of inducible nitric oxide synthase (iNOS) and cyclooxygenase-2 (COX-2) significantly (*p* < 0.05) increased in untreated groups (OA and OBOA) when compared to treated groups, as shown in Figure 6. As a result, the concentration of NO and prostaglandin E2 (PGE2) also increased in OA and OBOA groups. FJC supplementation significantly (*p* < 0.05) reduced these levels after being treated for 6 weeks. The OA and OBOA groups also showed high-level pro-inflammatory cytokines such as tumor necrosis factor (TNF)-α and leptin, as shown in Figure 7. The levels of TNF-α and leptin were significantly (*p* < 0.05) reduced after being treated with medium and high levels of FJC for 6 weeks of treatment.

### 3.5 Oral supplementation of FJC reduces matrix metalloproteinases and increases collagen type II levels

**FIGURE 8:**
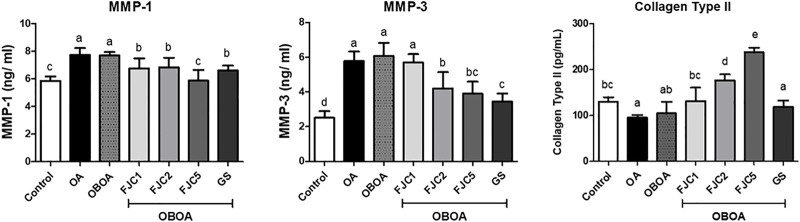
Effects of fermented jellyfish collagen (FJC) and glucosamine sulfate (GS) on matrix metalloproteinase (MMP)-1, MMP-3, and collagen type II level in rats’ plasma after treatment for 6 weeks. Different letters indicate (a–e) statistically different values (*p* < 0.05) between groups determined using one-way ANOVA with Duncan’s *post hoc* test.

The OA and OBOA groups showed high levels of matrix metalloproteinases MMP-1 and MMP-3, as shown in Figure 8. These levels were significantly (*p* < 0.05) reduced after being treated with medium and high doses of FJC (OBOA + FJC2 and OBOA + FJC5, respectively). These treatments also significantly (*p* < 0.05) increased collagen type II levels.

### 3.6 FJC supplementation protects against cartilage degradation

**FIGURE 9:**
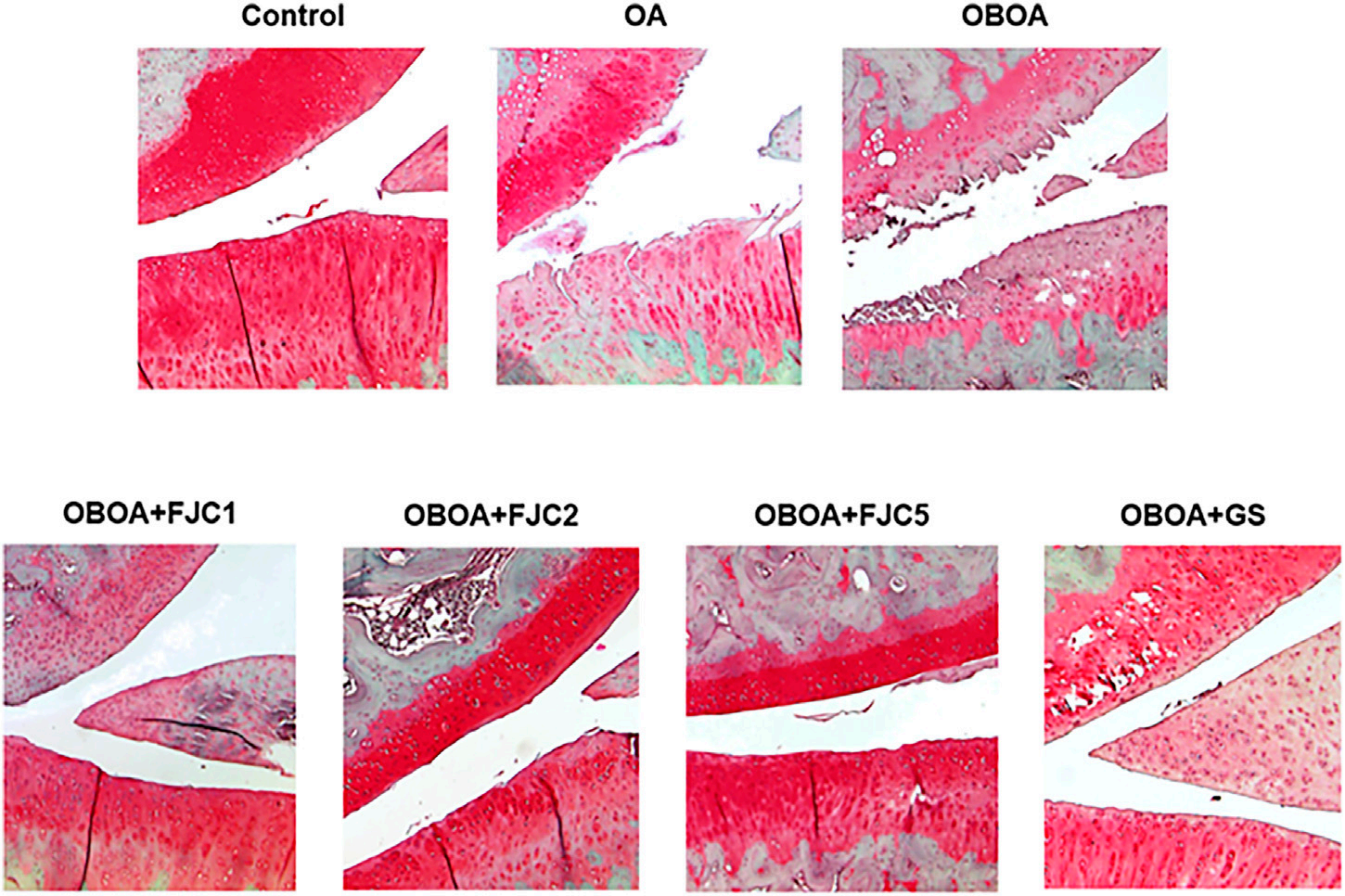
Representative of the Safranin O staining difference of the knee joint of each group after treatment for 6 weeks. Cartilage (orange to red) and nuclei (black).

Normal cartilage was observed in the control group, as shown in Figure 9. However, the OA and OBOA groups showed cartilage surface disruption. This condition also showed a high loss of proteoglycans, as indicated by the fading of the red color. After being treated by FJC for 6 weeks of treatment, the smooth cartilage surface was identified.

## 4 Discussion

In this present study, we successfully extracted and fermented the jellyfish (*Rhopilema esculentum*) using *Bacillus subtilis* natto to obtain fermented jellyfish collagen (FJC). A previous study reported that *B. subtilis* natto has been used for fermented collagen production (Hsu and Chiang, 2009). Additionally, a serine protease (nattokinase) and collagenase enzyme have been detected in the supernatant of *B. subtilis* culture (Nagano and To, 2014; Ju et al., 2019). The jellyfish’s collagen is composed of a high amount of glycine, proline, and hydroxyproline. These amino acids are the major amino acids (57%) in the collagen structure (Li and Wu, 2017). The FT-IR analysis showed five amide peaks: amide A, amide B, amide I, amide II, and amide III. A previous study reported that five amide peaks usually portray collagen characteristics (Seixas et al., 2020). The amide A band between 3,310 cm^−1^ and 3,270 cm^−1^ was associated with N–H stretching (Barth, 2007). Additionally, the band from 3,080 cm^−1^ to 2,889 cm^−1^ (amide B) was also related to N–H stretching (Riaz et al., 2018). The amide I band around 1,600 cm^−1^ indicated C = O stretching on proteins (de Campos Vidal and Mello, 2011), whereas N–H bending was represented by the amide II band (from 1,580 to 1,500 cm^−1^) (Murphy et al., 2014). The amide III band between 1,200 cm^−1^ and 1,350 cm^−1^ indicated C–N stretching and N–H bending (Cai and Singh, 2004).

An *in vivo* study demonstrated the ameliorative effects of FJC on surgically induced knee osteoarthritis (OA) in an obese rat model. A previous study also reported that cannonball jellyfish collagen reduced the pathogenesis of adjuvant arthritis in a rat model (Wood et al., 1969). The obesity condition has been considered an OA risk factor due to mechanical force in knee OA and proinflammatory production (Divella et al., 2016; Sudirman et al., 2022). Therefore, reduction of body weight or weight loss is a non-pharmacological OA treatment due to a decreased weight-bearing joint loading (Messier, 2008). Obesity is characterized by a high accumulation of adipose tissues or fats (Longo et al., 2019). The untreated OBOA group showed a high accumulation of fats, including epididymal and abdominal fats. This group also showed high levels of triglycerides (TG) and total cholesterol (TC). A previous study also reported that high levels of TG and TC were observed in obese conditions (Klop et al., 2013). The FJC showed anti-obesity properties by reducing the fat weight and TG and TC levels. Additionally, FJC treatment also increased the level of high-density lipoprotein cholesterol (HDL-C), whereas this level decreased in the obesity stage, as reported by a previous study (Wang and Peng, 2011). Previous studies also reported that marine collagen from the skin of skate (*Raja kenojei*) reduced the plasma LDL-C level and non-esterified free fatty acids (NEFAs) and increased the level of HDL-C. This marine collagen also downregulated the expression of fatty acid synthase (FAS) and acetyl-CoA carboxylase (ACC) in the animal model (Woo et al., 2018; Woo and Noh, 2020).

Low activity of enzymatic antioxidants such as superoxide dismutase (SOD) and glutathione peroxidase (GPx) was shown in OA and OBOA groups. Increasing the malondialdehyde (MDA) level was also observed in these groups. The MDA level was used as an indicator of the lipid peroxidation level and was also associated with an increase in free radical production (Mas-Bargues et al., 2021). In the case of surgically induced knee OA, oxidative stress also increased due to mechanical force (Yui et al., 2016). Also, oxidative stress was associated with an elevated level of obesity due to the presence of immoderate adipose tissue (Pottie et al., 2006). In the present study, we observed that the OBOA group shows a high level of oxidative stress, as indicated by the low activity of SOD and GPx and a high level of MDA. This condition due to obesity also increased free radical production. A previous study reported that obesity was observed in the reduction of SOD and GPx activities and was also associated with oxidative stress (Pandey et al., 2015; Masschelin et al., 2020). Additionally, the MDA level also increased in this condition (Jia et al., 2019). Oral supplementation of FJC showed antioxidant properties by enhancing enzymatic antioxidant activities and reducing MDA levels.

Nitric oxide (NO) is considered a proinflammatory mediator and is also associated with oxidative stress. This level increased in OA and OBOA groups. Increasing the NO level shows a positive correlation with the level of inducible nitric oxide synthase (iNOS). A previous study reported that iNOS synthesized NO from *l*-arginine and oxygen in chondrocytes (Ahmad et al., 2020). Additionally, chondrocyte apoptosis and cartilage degradation might be caused by NO produced in joints (Robinson et al., 2016). The level of prostaglandin-E2 (PG-E2) also increased in OA and OBOA groups. A high level of PG-E2 in the OA condition was associated with inflammation signs and progression, such as redness and pain. PG-E2 was also considered a proinflammatory mediator and was synthesized by cyclooxygenase (COX)-2 from arachidonic acid (Ricciotti and FitzGerald, 2011; Wang and DuBois, 2013). Additionally, COX-2 also was observed to increase in this present study. Previous studies also reported that the NO and PG-E2 levels also increased in the OA condition (Studer et al., 1999; Fermor et al., 2005). Some proinflammatory cytokines also were observed to be elevated in OA and OBOA groups, such as tumor necrosis factor-alpha (TNF-α) and leptin. A previous study reported that TNF-α and adipokines, such as leptin, are secreted by adipocytes and preadipocytes in the obese stage, and they indicated a state of chronic inflammation (Fernández-Sánchez et al., 2011). Additionally, elevated TNF-α and leptin are involved in the OA progression (Li et al., 2018; Yan et al., 2018). In the present study, fermented collagen from jellyfish shows anti-inflammatory activity by reducing some proinflammatory mediators and cytokines. A previous study also reported that marine collagen from skate reduced the leptin level in an animal model of obesity (Woo et al., 2018).

On the other hand, the p38/c-Jun N-terminal kinase (JNK) and mitogen-activated protein kinase (MAPK) pathways may be stimulated by inflammatory cytokines such as TNF-α to synthesize matrix metalloproteinase-1 (MMP-1) and MMP-3 (Vincenti and Brinckerhoff, 2002; Martin et al., 2003). Additionally, leptin also significantly induced MMP expression by activating AP-1 *via* the leptin receptor/MAPK/ERK signal transduction pathways (Liu et al., 2018). Therefore, the levels of MMP-1 and MMP-3 increased in the OA and OBOA groups. These enzymes are produced by synovial lining cells in arthritis conditions (Yoshihara et al., 2000). MMP-1, also known as collagenase-1, is a collagenase enzyme that is involved in the degradation of interstitial collagen (collagen types I, II, and III) and aggrecan (Burrage, 2006). Therefore, elevated MMP-1 is associated with cartilage degradation and OA progression (Wu et al., 2008). Additionally, MMP-3 could also be used as a potential biomarker for knee OA (Pengas et al., 2018). MMP-3, also known as stromelysin-1, is a neutral protease that is involved in the destruction of cartilage due to its capability of degrading some extracellular matrix components such as collagen (collagen types II, IX, X, and XI) and aggrecan (Naito et al., 1999). Therefore, inhibition of MMPs also is a key treatment for OA management due to cartilage composed of collagen (especially type II) and proteoglycans (especially aggrecan) (Fox et al., 2009). In this study, we observed that collagen type II increased after being treated with fermented jellyfish collagen (FJC) when compared to OA and OBAO groups. This condition indicated that FJC significantly protected collagen degradation during the treatment.

Under Safranin-O staining observation, we found that OA and OBOA groups show a high progression of OA development. According to a previous study, this staining result shows the nuclei (black), cartilage matrix (orange to red), and cytoplasm (bluish or gray-green). In this case, the loss of proteoglycans or cartilage is indicated by the loss of staining intensity (Schmitz et al., 2010). This condition may be caused by the activation of cartilage-degrading enzymes such as MMP-1 and MMP-3 by activation of the same transcription factor by proinflammatory cytokines (Pritzker et al., 2006). Additionally, oxidative stress is also involved in cartilage type II degradation (Henrotin et al., 2005; Pritzker et al., 2006). After being treated with FJC, we found that FJC treatment successfully protects cartilage degradation and suppresses OA development. A previous study reported that healthy cartilage is recognized by a smooth cartilage layer and associated chondrocytes present in well-ordered zones (Pritzker et al., 2006).

## 5 Conclusion

Fermented collagen from jellyfish (*Rhopilema esculentum*) showed the ameliorative effects of surgically induced osteoarthritis in obese rats. Oral supplementation of fermented collagen decreased body weight, triglyceride, and total cholesterol levels in obese rats. It also downregulated the expression of some proinflammatory cytokines, including tumor necrosis factor-α, cyclooxygenase-2, and nitric oxide; suppressed leptin and adiponectin expression; and attenuated cartilage degradation. Fermented jellyfish collagen also decreased the activities of matrix metalloproteinase (MMP)-1 and MMP-3. These results demonstrated that fermented jellyfish collagen showed a protective effect on articular cartilage and also suppressed the degradation of cartilage in an animal OA model, suggesting its potential efficacy as a promising candidate for OA treatment.

## Data Availability

The original contributions presented in the study are included in the article/Supplementary Material; further inquiries can be directed to the corresponding author.

## Abbreviations

COX, cyclooxygenase; FJC, fermented jellyfish collagen; GPx, glutathione peroxidase; JC, jellyfish collagen; MAPK, mitogen-activated protein kinase; MMP, matrix metalloproteinase; OA, osteoarthritis; OB, obesity; PG-E2, prostaglandin-E2; SOD, superoxide dismutase; TC, total cholesterol; TG, triglyceride.
